# Distinct cerebrovascular pathways underlying Alzheimer’s disease-related neurodegeneration

**DOI:** 10.1007/s00401-025-02970-8

**Published:** 2025-12-11

**Authors:** Rosaleena Mohanty, Sophia Wheatley, Konstantinos Chiotis, Anna Marseglia, Eric Westman

**Affiliations:** 1 Division of Clinical Geriatrics, Center for Alzheimer Research, Department of Neurobiology, Care Sciences and Society, Karolinska Institutet, Stockholm, Sweden; 2 The Ageing Epidemiology Research Unit, School of Public Health, Imperial College London, London, UK

**Keywords:** Cerebrovascular pathology, Arteriolosclerosis, Cerebral amyloid angiopathy, Alzheimer’s disease, Copathology, Neurodegeneration

## Abstract

The etiology of cerebrovascular pathology is heterogeneous. Independent or synergistic role of this pathology relative to Alzheimer’s disease (AD) pathology is necessary to clarify distinct neurodegenerative pathways. We evaluated the interplay of various cerebrovascular markers postmortem and their in vivo neuroimaging, clinical and neuropathologic correlates using data from the Alzheimer’s Disease Neuroimaging Initiative (ADNI). In 109 individuals, postmortem cerebrovascular pathology (atherosclerosis of the circle of Willis, cerebral amyloid angiopathy [CAA], arteriolosclerosis, white matter rarefaction, old infarcts, microinfarcts, hemorrhages, other ischemic/vascular changes) was characterized. Additionally, we assessed in vivo neuroimaging (cortical thickness, subcortical volume, white matter lesion burden, glucose standardized uptake value ratio, fractional anisotropy of white matter tracts, cerebral blood flow), cognitive, and neuropathologic measures (atrophy, AD pathology and copathologies including Lewy body, TDP-43, hippocampal sclerosis). The study sample had mean (standard deviation) age of 82.9 (7.2) years and included 29 women (27%) and 84 (77%) with intermediate/high AD neuropathologic change. Arteriolosclerosis and CAA emerged as dominant cerebrovascular markers using multiple correspondence analysis. More severe arteriolosclerosis was explained by higher white matter lesion burden and greater postmortem hippocampal atrophy (*β* = 143.2, 95% CI 63.9 to 230.1, *p* = 0.0003), but not AD pathology. More severe CAA was explained by fractional anisotropy (*β* = − 20, 95% CI − 41.5 to -3.1, *p* = 0.02) adjusted for AD pathology and reduced integrity of superior cerebellar peduncle, posterior thalamic radiation, and sagittal stratum tracts (*rho* < − 0.6, false discovery rate corrected *p* < 0.05). More severe CAA was also explained by cortical atrophy and AD pathology (*β* = 0.6, 95% CI 0.2 to 1.2, *p* = 0.007), and associated with poorer memory (*β* = − 0.2, 95% CI − 0.3 to -0.09, *p* = 0.0009). Results demonstrate two dominant cerebrovascular pathways. An arteriolosclerosis-driven pathway is unspecific to AD pathology, whereas a CAA-driven pathway is specific to AD pathology. Cerebrovascular pathology is associated with AD pathology in an etiology-dependent manner which may influence eligibility for treatment or treatment-emergent adverse events in disease-modifying therapies for AD.

**Supplementary Information:**

The online version contains supplementary material available at 10.1007/s00401-025-02970-8.

## Introduction

Cerebrovascular disease is common in the aging brain, can cause stroke, drive vascular dementia, and is a common comorbid contributor to mixed dementias. Risk factors commonly associated with sporadic cerebrovascular disease include age and vascular factors such as hypertension and diabetes mellitus [30, 62]. Clinical consequences of cerebrovascular disease can be serious including stroke, progressive cognitive impairment, gait and posture disorders, and depression, leading to loss of independence in daily life [55].

Cerebrovascular disease encapsulates pathologies with heterogeneous etiology including vessel injury (e.g., arteriolosclerosis, atherosclerosis, cerebral amyloid angiopathy) and eventually irreversible tissue injury (e.g., infarcts, hemorrhages) [2, 38]. These injuries are detectable radiologically [17, 63] or histologically [51, 57]. There is not a single in vivo marker fully capturing the extent/type of this pathology. Instead, markers may be derived from a combination of sequences (structural, diffusion, arterial spin labeling) [17]. Given the different markers and varied etiology, the relationship between in vivo and postmortem markers of cerebrovascular pathology remains to be fully investigated.

Considering Alzheimer’s disease (AD) which is the most common cause of dementia, cerebrovascular disease has been reported as a copathology in 33–75% of the brains assessed postmortem [9, 35]. This is especially relevant for Aβ-lowering disease modifying immunotherapies in AD where cerebrovascular markers (e.g., microhemorrhages) can inform the risk of developing treatment-emergent adverse events such as amyloid-related imaging abnormalities (ARIA) [27]. Recognizing the contribution of comorbid pathologies [31], AD brains also commonly have Lewy body pathology [6], hippocampal sclerosis [10], and TAR DNA-binding protein 43 (TDP-43) [7]. Across both AD and non-AD dementias, cerebrovascular pathology has been shown to increase over age [1]. How these copathologies relate to cerebrovascular markers is yet another open question.

Thus, in this study, we investigated three questions in the context of AD: (1) Which are the dominant markers of cerebrovascular pathology? (2) Which are the in vivo, clinical, and neuropathologic correlates of the dominant cerebrovascular markers? (3) What are the possible mechanisms involving the dominant cerebrovascular markers in relation to neurodegeneration? We hypothesized that contribution of individual cerebrovascular markers would differ in relation to other cerebrovascular markers, AD markers, and neurodegeneration. Identifying key cerebrovascular markers and associated pathways could better inform the search for useful biomarkers for clinical trials, given the relevance of cerebrovascular pathology in recent disease-modifying trials for AD.

## Materials and methods

### Participants

We selected all available cases (110 individuals) from the Alzheimer’s Disease Neuroimaging Initiative (ADNI; RRID:SCR_003007) who underwent postmortem neuropathologic examination and included available in vivo neuroimaging and clinical data as of May 2024. The ADNI was launched in 2003 as a public–private partnership, led by Principal Investigator Michael W. Weiner, MD. The primary goal of the ADNI has been to test whether serial magnetic resonance imaging (MRI), positron emission tomography (PET), other biological markers, and clinical and neuropsychological assessment can be combined to measure the progression of mild cognitive impairment (MCI) and early AD as per www.adni-info.org. In general, individuals aged 55–90 years across cognitively normal, MCI and mild AD dementia groups were enrolled in the ADNI as per the inclusion criteria. Individuals with any significant neurologic disease other than AD were excluded. Of relevance to the current study, individuals showing evidence of infarction, focal lesions, or lacunes at baseline MRI were excluded. We selected individuals who consented to brain donation and underwent histopathologic assessment of postmortem biomarkers based on National Alzheimer's Coordinating Center (NACC) Neuropathology forms (version 10 and 11). Subsample of individuals had MRI (T1 weighted, diffusion tensor imaging, arterial spin labeling sequences) or FDG PET acquired at the time point closest to death which were also included. Inclusion/exclusion criteria and methodologies for neuropathologic, neuroimaging, and clinical data are documented and available at: https://adni.loni.usc.edu/help-faqs/adni-documentation/. All individuals provided written informed consent prior to participation in study procedures in accordance with the Declaration of Helsinki, and approval for the study was obtained by the local ethics committees of each participating site within ADNI. Procedures to obtain informed consent for postmortem assessment were followed as previously established [12]. We followed the Strengthening the Reporting of Observational Studies in Epidemiology (STROBE) reporting guideline.

### Image acquisition and processing

#### MRI

T1-weighted MRI were acquired on 1.5 T or 3 T scanners with sagittal 3D magnetization-prepared rapid gradient-echo sequences. MRI were pre-processed in-house [44] using cross-sectional FreeSurfer stream v6.0.0 (https://freesurfer.net/). Resulting segmentations were visually examined for quality control. Automatic region of interest parcellation yielded thickness of cortical structures and volumes of deep/subcortical structures [16, 20] measuring gray matter integrity. White matter hypointensity volume was used as an in vivo surrogate for cerebrovascular pathology [13, 41, 48]. All volume measures were normalized for estimated intracranial volume. Additionally, we examined AD signature thickness as surface-area weighted average of mean cortical thickness in the entorhinal, inferior temporal, middle temporal, and fusiform regions [32].

Diffusion tensor images (DTI) were acquired with 46 separate images including 5 T2-weighted images with no diffusion sensitization (b0 images) and 41 diffusion-weighted images (*b* = 1000 s/mm^2^). Processed DTI were obtained directly from ADNI which were screened for quality and pre-processed using FSL (www.fmrib.ox.ac.uk/fsl). Global and regional mean of fractional anisotropy in 40 bilateral white matter tracts of the JHU DTI-based white matter atlas [43] measured white matter microstructural integrity.

Arterial spin labeling (ASL) images were acquired with 3 T scanners and comprised 2D pulsed ASL (ADNI 2), 3D pulsed ASL, or 3D pseudo-continuous ASL (ADNI 3). Processed ASL were obtained directly from ADNI and included quality control, motion correction, perfusion-weighted image computation, geometric distortion and partial volume corrections, cerebral blood flow computation using a combination of FreeSurfer, FSL, SPM8 (http://www.fil.ion.ucl.ac.uk/spm), Insight Toolkit (ITK), and MATLAB. Global and regional mean cerebral blood flow was computed in the same brain regions as provided by FreeSurfer [16, 20] as a quantitative measure of blood flow in the capillary bed in the brain tissue.

#### FDG PET

FDG PET included dynamic 3D scans made up of six 5-min frames retrieved 30–60 min after administration of [18F] FDG. Processed FDG PET output was directly obtained from ADNI where coregistered and standardized images were spatially normalized to MNI PET template using SPM. Standardized uptake ratio (SUVR) scaled to pons/vermis as reference region was extracted for AD signature region (mean across bilateral angular gyrus, posterior cingulate, inferior temporal gyrus [39]). Additionally, SUVR was generated for regions provided by FreeSurfer [16, 20] using PETSurfer [25, 26].

### Cognitive outcomes

We examined global cognition by Mini-Mental State Examination (MMSE) and cognitive domains by composite scores for memory, executive function, language, and visual–spatial abilities [45].

### Postmortem neuropathology

Neuropathologic assessment of AD [42] was conducted following the NIA-AA guidelines within ADNI’s neuropathology core [22]. To analyze cerebrovascular pathology, we selected eight available markers: (1) atherosclerosis of the circle of Willis, (2) arteriolosclerosis in the subcortical white or gray matter, (3–6) old gross infarcts, old gross hemorrhages, old microinfarcts, or other pathologic changes related to ischemic or vascular disease (laminar necrosis, acute infarcts, vascular malformation, aneurysm, vasculitis, CADASIL, or mineralization of blood vessels) in the minimum recommended brain regions, (7) cerebral amyloid angiopathy (CAA) in the parenchymal, and/or leptomeningeal vessels in the minimum recommended brain regions, and (8) white matter rarefaction in the white matter pallor in the centrum semiovale and subcortical white matter. Minimum recommended brain regions [42] are medulla including dorsal motor nucleus of the vagus, pons including locus ceruleus, midbrain including substantia nigra, cerebellar cortex and dentate nucleus, thalamus and subthalamic nucleus, basal ganglia at the level of the anterior commissure with the basal nucleus of Meynert, hippocampus and entorhinal cortex, cingulate, anterior, amygdala, middle frontal gyrus, superior and middle temporal gyri, inferior parietal lobule, occipital cortex (Brodmann area 17 and 18), white matter at the anterior cerebral artery, middle cerebral artery, and posterior cerebral artery watershed. One participant was excluded due to missing values across multiple markers (three of eight including atherosclerosis of the circle of Willis, old gross infarcts including lacunes, old hemorrhages), leading to a final sample of 109 participants. We evaluated core AD pathologies by Thal stage, Braak stage, Consortium to Establish a Registry for Alzheimer's Disease (CERAD) score, and AD neuropathologic change; neurodegeneration by cortical and hippocampal atrophy; and non-AD copathologies by Lewy body pathology, TDP-43, hippocampal sclerosis. Neuropathologic variables were semi-quantitative and ordinal. Specifically, atherosclerosis, arteriolosclerosis, CAA, and white matter rarefaction were rated on a 4-point scale (none, mild, moderate, severe), while old gross infarcts, old gross hemorrhages, old microinfarcts, and other pathologic changes related to ischemic or vascular disease were binary and rated as either being present or absent.

### Statistical analysis

To address our first question of examining the simultaneous relationship among the postmortem cerebrovascular markers to identify the dominant ones, we employed multiple correspondence analysis. This analysis offers an unsupervised data reduction approach for semi-qualitative variables [58]. Multiple correspondence analysis (RRID:SCR_014602) was used to model the eight cerebrovascular markers and simplify the model by minimizing the number of variables without a priori information about the correlation between variables to facilitate easier interpretation. Maximizing contribution of all variables, multiple correspondence analysis allows to model the variance from missing values after ruling out potential outliers. Missing data was treated as a separate factor level and treated as its own group in this model, which enables to understand how the missing group relates to other variables or categories in the multiple correspondence space without ignoring whole observations due to missing data only in a subset. The model is helpful in identifying a smaller number of uncorrelated axes describing the spread of data. To address our second question of identifying the correlates, we investigated the key axes identified by multiple correspondence analysis further by correlating them with neuroimaging markers (MRI, FDG PET, DTI, ASL) and cognitive (MMSE, cognitive domains) and neuropathologic variables (AD markers, atrophy, non-AD copathologies) using the Spearman’s partial correlation model [15] (correlation coefficient, *rho*). We reported *p*-values after controlling for false discovery rate (FDR), and *p*_*FDR*_ < 0.05 was deemed significant. Based on the identified correlates, we addressed our third question of possible mechanisms related to the key axes identified by multiple correspondence analysis using regression analysis—ordinal logistic [28] and multiple linear regression for ordinal and continuous outcomes, respectively. In these conventional models computing partial correlations and regressions, missing data was handled by only including complete cases and dropping missing observations. The following covariates were included in the partial correlation and regression analyses: age at death if only postmortem data were included, difference in age (i.e., scan-to-postmortem examination interval) if both neuroimaging/cognitive and postmortem data were included, education for cognitive data, and relevant risk factors based on prior literature [8, 47, 53, 65] and availability of data (history of hypertension or *APOE* ɛ4 carriership). Analyses were performed in R v4.4.2 (RRID:SCR_000432).

## Results

**Fig. 1:**
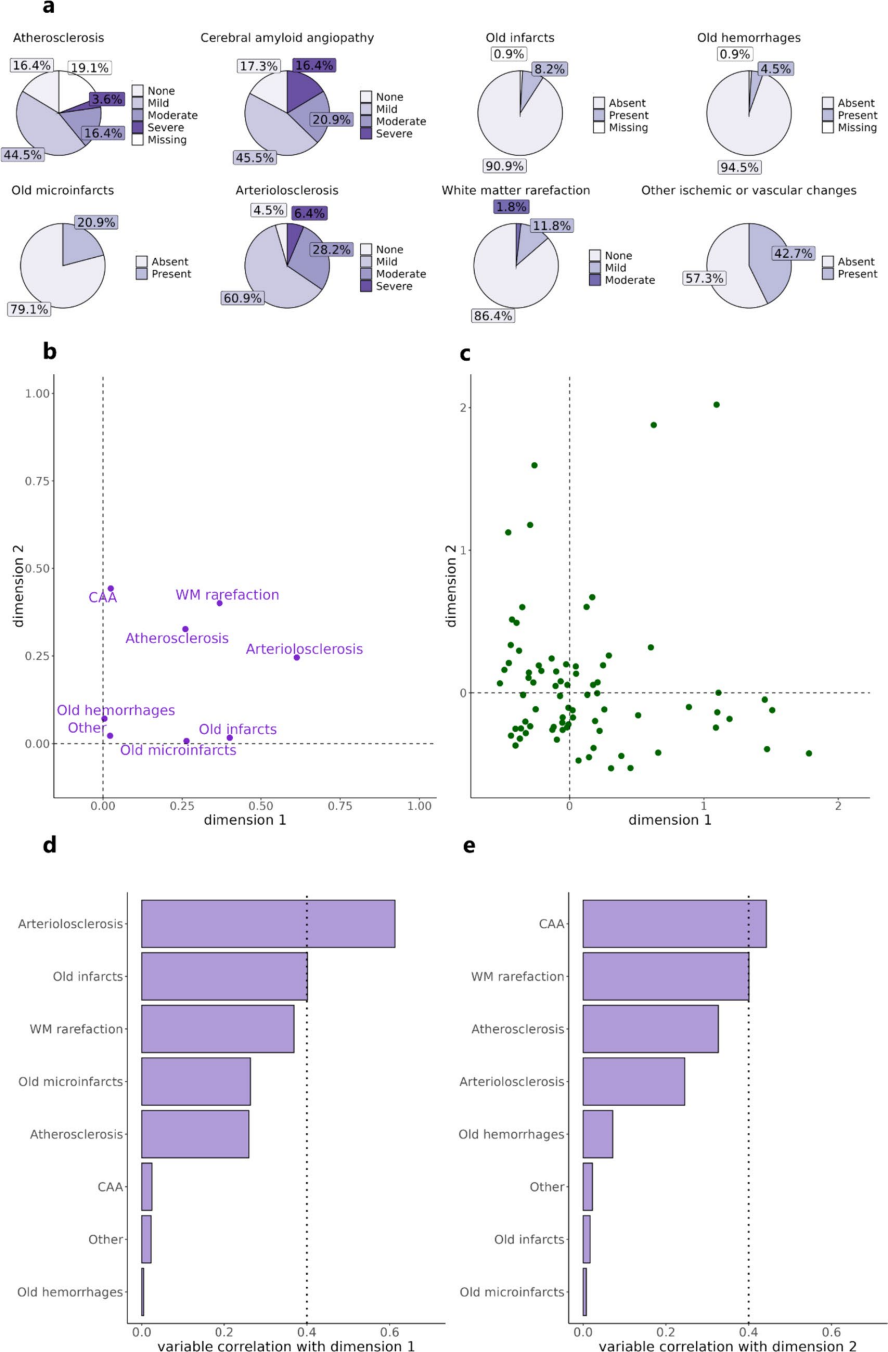
Distribution and relationship of the cerebrovascular markers. **a** Distribution of eight cerebrovascular markers in the study sample. **b** Two-dimensional representation obtained from multiple correspondence analysis which models the relationship among the eight cerebrovascular markers. **c** Distribution of individuals along the two dimensions obtained from multiple correspondence analysis. **d** Correlation of each cerebrovascular marker with dimension 1 and **e** dimension 2. Moderate correlation = 0.4 of each marker to the dimensions was considered as threshold for comparison (dotted line). *CAA* cerebral amyloid angiopathy, *WM* white matter

Demographics, clinical, and neuropathologic characteristics of study participants are presented in Table 1. The study sample was aged 83 ± 7 years at death, with 58% being *APOE* ɛ4 carriers, impaired in cognition (MMSE = 20 ± 7). Age difference (or interval) between neuroimaging-to-postmortem evaluations was systematically higher than that between neuroimaging-to-death. We note a high frequency of intermediate/high AD neuropathologic change postmortem (77%). Aside from the pathologies listed in Table 1, we did not stratify or adjust for other pathological subtypes due to missing data and/or limited variability (e.g., frontotemporal lobar degeneration with TDP-43 pathology was seen in two cases in the full sample, while frontotemporal lobar degeneration with tau pathology had 53% missing data and included two cases with progressive supranuclear palsy and none with corticobasal degeneration). Distribution of the eight cerebrovascular markers assessed postmortem is shown in Fig. 1a. All individuals showed the presence of at least one of these cerebrovascular markers. The majority (about ≥ 50%) showed some degree of atherosclerosis, CAA, arteriolosclerosis, or other vascular changes. The minority (about ≤ 20%) had evidence of white matter rarefaction, old gross infarcts, microinfarcts, and hemorrhages. Old cerebral microbleeds were absent in all participants and were, thus, excluded from analysis due to lack of variability. We evaluated ≥ 3 mm infarcts based on MRI (time from postmortem examination = 6.0 ± 3.5 years) detected by a trained expert directly available in ADNI. In this sample, 15 (13.8%) individuals showed MRI infarcts, localized in the thalamus (*n* = 5, one was hemorrhagic), basal ganglia (*n* = 3), cerebellum (*n* = 3), frontal cortex (*n* = 1), frontal white matter (*n* = 1), internal capsule (*n* = 1), and temporal white matter (*n* = 1, hemorrhagic). All others were thrombotic infarcts. About half of these cases (*n* = 7) showed the presence of one or more infarcts in neuropathologic examination (Supplementary Table 1).

**Table 1:** Demographic, clinical, and neuropathologic characteristics of the study participants

Variable	*N*	Value
Age at death (years)	109	82.9 ± 7.2
Sex (*N*, % women)	109	29 (27%)
Education (years)	109	16.2 ± 2.8
Ethnicity	109	96% White, 3% Black or African American, 1% Asian
*Risk factors*		
*APOE* ɛ4 carriers (*N*, %)	109	63 (58%)
History of hypertension (*N*, % present)	109	63 (57%)
*Cognition*		
Mini-Mental State Examination score	109	19.9 ± 6.8
Memory composite	109	− 0.9 ± 0.9
Executive function composite	109	− 0.6 ± 0.9
Language composite	109	− 0.5 ± 0.8
Visuospatial ability composite	109	− 0.5 ± 0.7
*In vivo neuroimaging*		
MRI-to-death interval (years)	109	3.2 ± 2.8
FDG PET-to-death interval (years)	88	3.9 ± 2.7
DTI-to-death interval (years)	20	2.6 ± 1.5
ASL-to-death interval (years)	14	3.1 ± 2.5
MRI-to-postmortem examination interval (years)	109	5.3 ± 3.5
FDG PET-to-postmortem examination interval (years)	88	5.7 ± 3.4
DTI-to-postmortem examination interval (years)	20	4.5 ± 1.9
ASL-to-postmortem examination interval (years)	14	5.1 ± 3.2
*Neuropathologic findings*		
Thal stage (*N*, % > Phase 2)	109	96 (88%)
Braak stage (*N*, % > Stage II)	109	86 (79%)
CERAD score (*N*, % > sparse)	109	74 (68%)
AD neuropathologic change (*N*, % intermediate/high)	109	84 (77%)
Lewy body (*N*, % present)	109	54 (50%)
TDP-43 (*N*, % present)	98	49 (55%)
Hippocampal sclerosis (*N*, % present)	109	10 (9%)
Cortical atrophy (*N*, % present)	92	83 (90%)
Hippocampal atrophy (*N*, % present)	94	80 (85%)

*APOE* ɛ4, ε4 allele of the apolipoprotein E, *MRI* magnetic resonance image, *FDG PET* fluorodeoxyglucose positron emission tomography, *DTI* diffusion tensor image, *ASL* arterial spin label, *AD* Alzheimer’s disease, *CERAD* Consortium to Establish a Registry for Alzheimer’s Disease, *TDP-43* TAR DNA-binding protein 43 (TDP-43)

### Arteriolosclerosis and CAA emerge as dominant among cerebrovascular markers

Firstly, we assessed the dominant cerebrovascular markers upon accounting for the interplay of the eight cerebrovascular markers using multiple correspondence analysis. Figure 1b-c shows the relationship of the eight cerebrovascular markers and distribution of participants along two dimensions. Two dimensions were chosen based on elbow method [4] (Supplementary Fig. 1) and showed a cumulative variance of 22%. We evaluated cerebrovascular markers showing moderate to strong correlation (> 0.4) with each dimension—arteriolosclerosis for dimension 1 (Fig. 1d; correlation = 0.61, *p* < 0.0001) and CAA for dimension 2 (Fig. 1e; correlation = 0.44, *p* < 0.0001).

### Arteriolosclerosis and CAA have distinct correlates

**Fig. 2:**
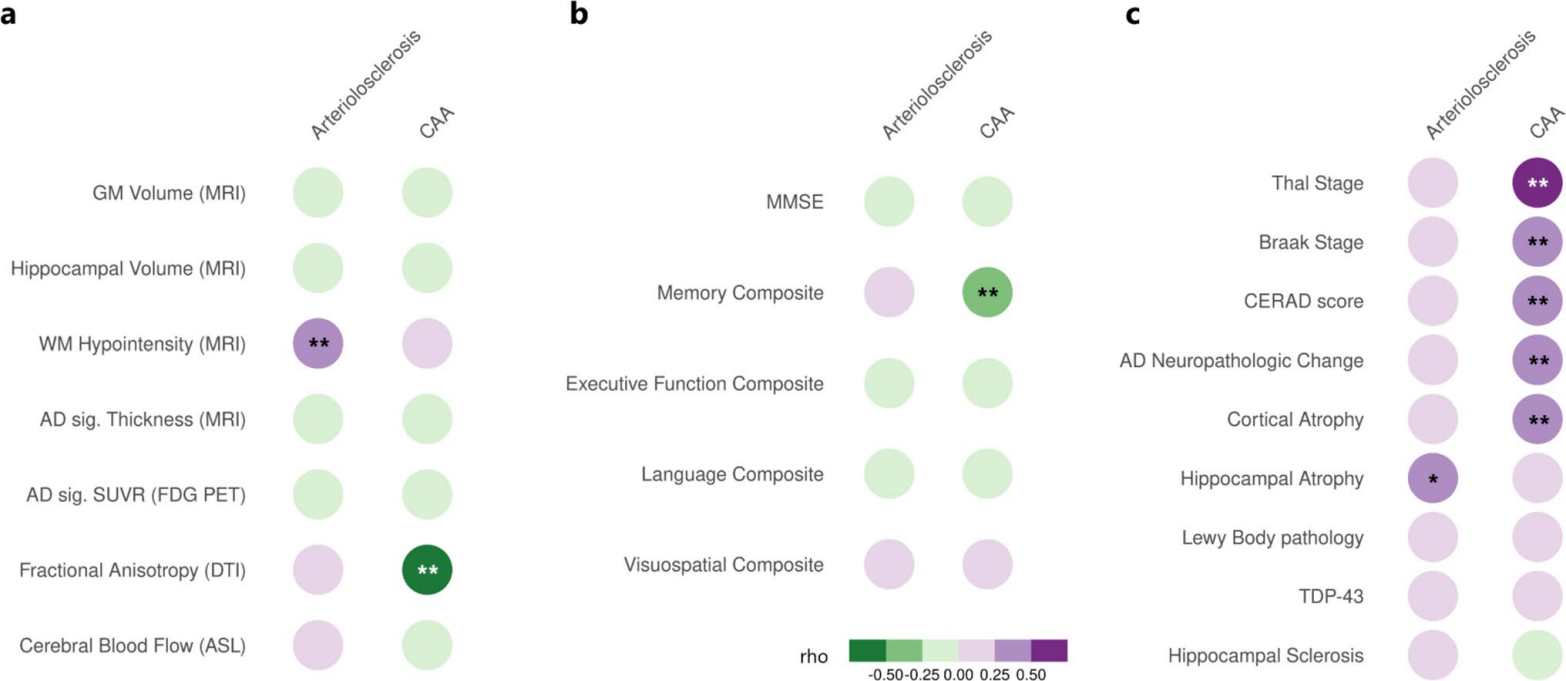
Correlates of arteriolosclerosis and cerebral amyloid angiopathy. Correlates of arteriolosclerosis and cerebral amyloid angiopathy with **a** in vivo neuroimaging measures based on MRI, FDG PET, DTI, ASL, **b** global and domain-specific cognitive scores (education-adjusted), and **c** postmortem AD and non-AD neuropathologic assessments. Partial correlations (rho) were visualized. Significant results indicated by ** correspond to *p*_FDR_ < 0.05 and marginally significant result indicated by * corresponds to *p*_FDR_ = 0.06. *CAA* cerebral amyloid angiopathy, *MRI* magnetic resonance imaging, *GM* gray matter, *WM* white matter, *FDG PET* [^18^F]fluorodeoxyglucose positron emission tomography, *AD* Alzheimer’s disease, *AD sig.* Alzheimer’s disease signature, *DTI* diffusion tensor imaging, *ASL* arterial spin labeling, *MMSE* Mini-Mental State Examination, *CERAD* Consortium to Establish a Registry for Alzheimer’s Disease, *TDP-43* TAR DNA-binding protein 43 (TDP-43)

Secondly, we investigated in vivo, clinical and neuropathologic correlates of the dominant cerebrovascular markers using partial correlation models. Among in vivo neuroimaging correlates (Fig. 2a), arteriolosclerosis was associated with white matter hypointensity volume (*rho* = 0.30, *p*_FDR_ = 0.03), while CAA was associated with mean fractional anisotropy (*rho* = − 0.55, *p*_FDR_ = 0.049). We observed that neither total white matter volume (*rho* = − 0.06, *p* = 0.47) nor total white matter hypointensity volume (*rho* = 0.16, *p* = 0.08) was significantly associated with white matter rarefaction. Among cognitive correlates (Fig. 2b), CAA but not arteriolosclerosis was associated with memory composite score (*rho* = − 0.31, *p*_FDR_ = 0.006). Among postmortem correlates (Fig. 2c), we only observed a marginally significant association between arteriolosclerosis and hippocampal atrophy (*rho* = 0.3, *p*_FDR_ = 0.06). However, CAA was correlated with Thal stage (*rho* = 0.52, *p*_FDR_ < 0.001), Braak stage (*rho* = 0.41, *p*_FDR_ < 0.001), CERAD score (*rho* = 0.45, *p*_FDR_ < 0.001), AD neuropathologic change (*rho* = 0.40, *p*_FDR_ < 0.001), and cortical atrophy (*rho* = 0.25, *p*_FDR_ = 0.049).

### Differential association of arteriolosclerosis and CAA with neurodegeneration

**Fig. 3:**
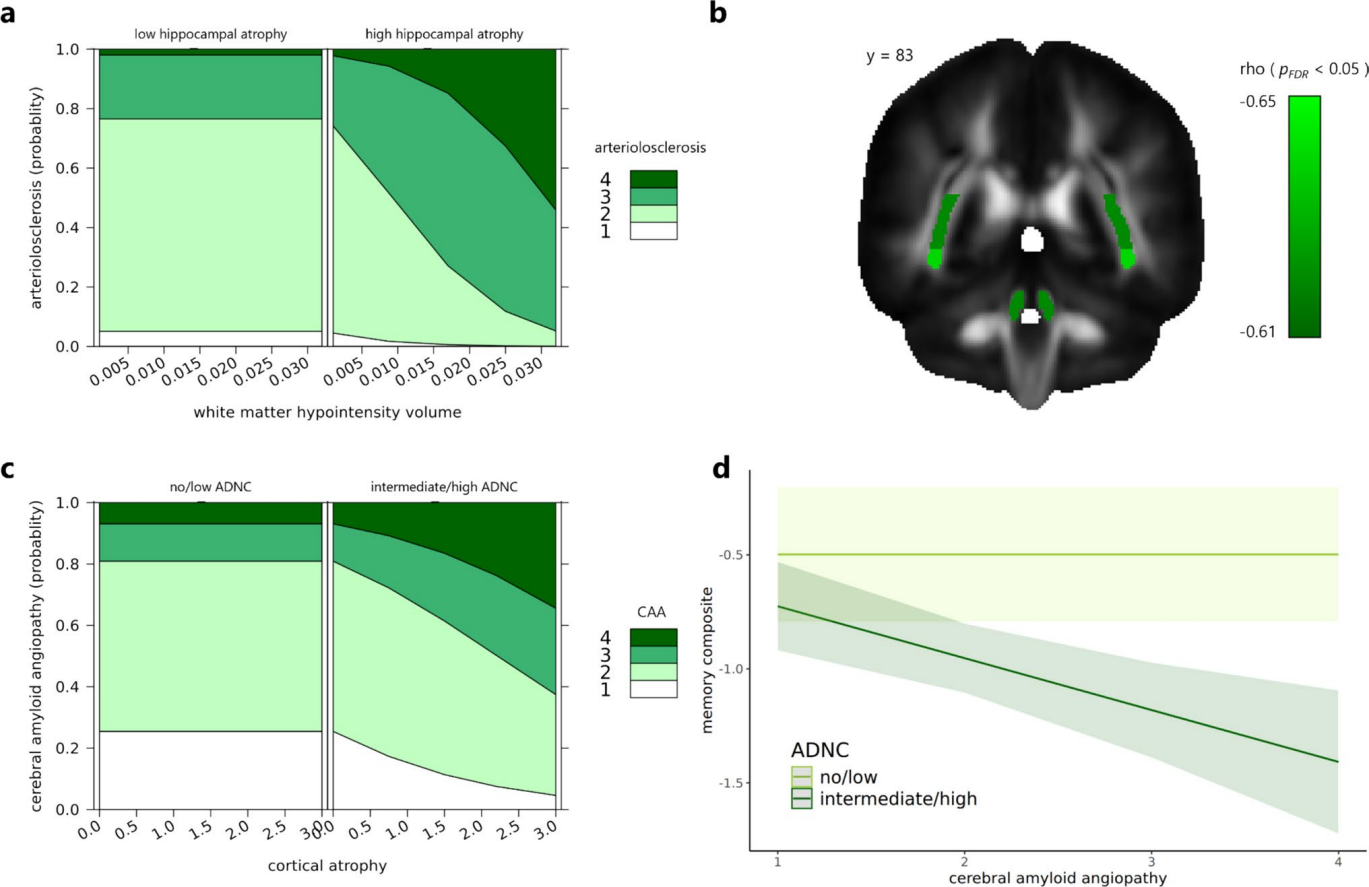
Possible mechanisms explaining the severity of arteriolosclerosis and cerebral amyloid angiopathy. **a** Severity of arteriolosclerosis was explained by a significant interaction of white matter hypointensity volume and hippocampal atrophy. White matter hypointensity volume is normalized by the estimated intracranial volume. Hippocampal atrophy was binarized as low (none or mild) or high (moderate or severe). The model was adjusted for difference in age between in vivo MRI and postmortem examination, and history of hypertension as a risk factor. **b** More severe CAA was associated with decreased fractional anisotropy of three white matter tracts including superior cerebellar peduncle, posterior thalamic radiation, and sagittal stratum. Fractional anisotropy values were averaged across hemispheres. Partial correlation for each tract is represented by rho for *p*_FDR_ < 0.05. **c** Severity of cerebral amyloid angiopathy was explained by a significant interaction of cortical atrophy and ADNC status. The model was adjusted for age at death and APOE ɛ4 carriership as a risk factor. **d** Memory scores were explained by a significant interaction of cerebral amyloid angiopathy and ADNC status. The model was adjusted for difference in age between cognitive and postmortem examinations, education, and APOE ɛ4 carriership as a risk factor. Levels of arteriolosclerosis, CAA, and cortical atrophy include 1 = none, 2 = mild, 3 = moderate, and 4 = severe. *CAA* cerebral amyloid angiopathy, *ADNC* Alzheimer’s disease neuropathologic change

Thirdly, we analyzed possible neurodegenerative mechanisms of cerebrovascular disease based on previous analyses. A significant two-way interaction in ordinal logistic regression indicated that individuals with higher in vivo white matter hypointensity volume and postmortem hippocampal atrophy showed more severe arteriolosclerosis (Fig. 3a; Supplementary Table 2: *β* = 143.2, 95% CI 63.9 to 230.1, *p* = 0.0003). Decreased global mean fractional anisotropy was significantly associated with more severe CAA seen in ordinal logistic regression (Supplementary Table 3: *β* = − 20, 95% CI − 41.5 to -3.1, *p* = 0.02). Further regional analyses using partial correlation showed that CAA severity was associated with decreased mean fractional anisotropy in three tracts (Fig. 3b) including superior cerebellar peduncle (*rho* = − 0.62, *p*_FDR_ = 0.03), posterior thalamic radiation (*rho* = − 0.62, *p*_FDR_ = 0.03), and sagittal stratum (*rho* = − 0.64, *p*_FDR_ = 0.03). A significant two-way interaction in ordinal logistic regression indicated that individuals with intermediate/high ADNC and greater cortical atrophy postmortem showed more severe CAA (Fig. 3c; Supplementary Table 4: *β* = 0.6, 95% CI 0.2 to 1.2, *p* = 0.007). Further multiple linear regression showed that individuals with intermediate/high ADNC and more severe CAA exhibited poorer memory scores (Fig. 3d; Supplementary Table 5: *β* = − 0.2, 95% CI − 0.3 to − 0.09, *p* = 0.0009). Regional correlates of arteriolosclerosis and CAA based on MRI, FDG PET, and ASL did not survive multiple comparison corrections (Supplementary Fig. 2).

## Discussion

Using a data-driven approach, we investigated the relationship among eight cerebrovascular markers postmortem. As hypothesized, some cerebrovascular markers are more closely linked to markers of AD than others. Two uncorrelated pathways emerged after accounting for inter-relationship among cerebrovascular markers with distinct imaging and clinical and pathologic correlates. One pathway was driven by arteriolosclerosis and associated with in vivo white matter lesion burden and postmortem hippocampal atrophy reflecting a non-AD-specific mechanism. A second pathway was driven by CAA and associated with poor in vivo white matter microstructural integrity, poor memory, postmortem cortical atrophy, and core AD hallmarks, reflecting an AD-specific mechanism. Interestingly, both arteriolosclerosis and CAA are major causes of small vessel disease [5, 30, 51] and represent vessel wall injury. Our findings added insights that parenchymal injury associated with each form of vessel wall injury is distinct.

Cerebrovascular pathology is increasingly being recognized as a driving or comorbid pathology in neurodegenerative diseases [31, 35]. There is ongoing debate on independent versus synergistic relationship of AD and cerebrovascular pathologies [3, 36, 37]. We observed that 77% of the sample had intermediate/high AD neuropathologic change (confirmed AD diagnosis), while incidence of cerebrovascular pathology ranged from 4.5 to 95.5% depending on the marker type. Heterogeneous manifestation of cerebrovascular pathology has been reported in vivo [17]^7^ and postmortem [57]^9^, making it challenging to disentangle its mechanisms from AD pathology. In a sample with limited cerebrovascular burden, we show here that the association between AD and cerebrovascular pathologies may be dependent on the etiology—arteriolosclerosis-related neurodegeneration did not vary by AD pathology, whereas CAA-related neurodegeneration was exacerbated for higher AD pathology.

Arteriolosclerosis is characterized by loss of smooth muscle cells, deposits of fibro-hyaline material, narrowing of the lumen, or vessel wall thickening of arterioles or arteries [51, 57]. Firstly, we found that more severe arteriolosclerosis was linked to higher in vivo white matter lesion burden. An explanation could be that arteriolosclerosis is most common in hypertensive individuals [29, 34] and white matter lesions can be induced due to hypertension [50]. Secondly, we found that more severe arteriolosclerosis was marginally associated with hippocampal atrophy postmortem. This may be possibly explained by the hippocampal sclerosis-aging pathogenesis hypothesis which posits a close link among systemic factors (e.g., hypertension), arteriolosclerosis, and hippocampal sclerosis [47]. Individuals with hippocampal sclerosis showed arteriolosclerosis in several cortical and subcortical regions, suggesting a brain-wide pathological process. It has been reported that hippocampal atrophy driven by hippocampal sclerosis, often overlapping with TDP-43 [46], is independent of AD pathology [49]. Third and importantly, we noted a significant interaction of in vivo white matter lesion burden and postmortem hippocampal atrophy explaining the severity of arteriolosclerosis. Interaction between white matter lesion burden and medial temporal atrophy has been reported in non-AD dementia [54]. Altogether, these findings may suggest that parenchymal injury associated with arteriolosclerosis follows a non-AD specific pathway—hippocampal atrophy may be partly attributed to Wallerian degeneration [18] from hypertension-induced white matter lesions and/or to non-AD pathologies such as hippocampal sclerosis.

CAA is characterized by progressive deposition of Aβ protein in the walls of arteries and arterioles in the leptomeningeal space, the cortex, and possibly in the capillaries and veins [51, 64]. Firstly, the severity of CAA was associated with decreased in vivo white matter microstructural integrity globally and regionally in three tracts including superior cerebellar peduncle, posterior thalamic radiation, and sagittal stratum. This finding is in line with prior evidence showing reduced integrity in the posterior tracts in Aβ + individuals with probable CAA compared to AD [33]. Stereotypical spread of CAA progresses from leptomeningeal and neocortical vessels to the medial temporal lobe and cerebellar vessels to the basal ganglia and thalamus [60]. White matter tracts showing compromised integrity in our findings align well with the advanced stages of CAA. A plausible mechanism could be that deposition of Aβ weakens vessel walls, disrupting arterioles which supply the deep white matter [40]. Secondly, CAA correlated with core AD hallmarks (Aβ, tau, CERAD, AD neuropathologic change). Of the cases with confirmed AD, 33% had advanced CAA (moderate–severe levels corresponding with substantial pathology in parenchymal and/or leptomeningeal vessels) within the estimated range of 25–44% for co-occurrence from prior studies [19, 65]. Despite shared involvement of Aβ in AD and CAA, they represent distinct entities [24]. Lastly, we observed a significant interaction such that advanced CAA and confirmed AD neuropathologic change explained poorer memory scores and postmortem cortical atrophy (Fig. 3d). This result suggests that co-occurring pathologies in AD can alter biological and clinical outcomes consistent with the revised criteria for AD [31], although caution should be exercised not to treat this result as a causal finding given the cross-sectional nature of our analysis. Regarding CAA-related cortical neurodegeneration, possible mechanisms may arise from concomitant parenchymal AD pathology, cortical effects of CAA-related structural lesions, or CAA-related vascular dysfunction [21]. Regarding CAA-related cognitive outcomes, it has been shown that interaction of advanced parenchymal Aβ in AD and vascular Aβ in CAA contribute to cognitive decline through tau pathology [52]. This interaction speaks to the synergistic interplay of cerebrovascular (CAA) and neurodegenerative (AD) pathologies in determining pathological and clinical deficits in AD. Taken together and in contrast to the mechanisms related to arteriosclerosis, parenchymal injury associated with CAA follows an AD-specific pathway.

Findings from this study have important implications in the context of Aβ-lowering disease modifying immunotherapies, as the mechanisms underlying the adverse events are closely related to brain vasculature, seen as ARIA [59]. It is currently recommended to exclude individuals with pre-existing cerebrovascular disease for treatment, particularly if there exist markers of CAA or ischemic brain injury [23]. However, risk factors for ARIA include *APOE* ε4 carriership and hypertension [11, 56, 61], which are key risk factors for CAA and arteriolosclerosis, respectively. The two pathways in this study may have differing implications for the approved treatments. CAA may help assess the risk of developing ARIA in individuals undergoing treatment, whereas arteriolosclerosis may help identify individuals at a higher risk of developing new infarcts, screening out individuals ineligible for treatment. Specific correlates of CAA (white matter microstructure and cortical atrophy adjusted for *APOE* ε4 carriership) and arteriolosclerosis (white matter lesion and hippocampal atrophy adjusted for hypertension) could be potential candidates for screening of individuals at risk for developing ARIA, thereby improving future clinical trial design. Such markers identified based on postmortem validation are necessary to develop reliable tools to maximize therapeutic benefits and minimize potential risks.

This study has some limitations. The overall sample size was modest, and subsamples based on DTI (*n* = 20) and ASL (*n* = 14) imaging were especially small, limiting the statistical power. Thus, the results based on these small samples should be treated with caution. However, there exist very few cohorts with both imaging sequences and postmortem data. While results should be reliable as they were evaluated against gold standard postmortem examination, further replication in independent cohorts is warranted. Some cerebrovascular markers were evaluated for presence or absence (especially markers of tissue injury such as infarcts, hemorrhages) and information about their severity would have been useful. Cerebral microbleeds constitute another cerebrovascular marker, which was absent postmortem in all individuals and due to lack of variability could not be assessed in this study. This may be explained by methodological considerations such as assessment of a limited number of sections of the cerebral cortex, subcortical white matter and periventricular white matter, subcortical gray matter, brainstem, and cerebellum. Microbleeds are closely related to CAA [14, 30] and should be investigated further. To identify dominant cerebrovascular markers, we considered two dimensions for ease of interpretation. Interaction of multiple cerebrovascular markers per dimension may be worth considering. Individuals with baseline evidence for infarctions, lacunes, and stroke or higher vascular risk were excluded in ADNI, which can underestimate cerebrovascular burden in the sample. Despite this limitation, we may still expect some cerebrovascular pathology to develop over time in older brains given that ADNI is a longitudinal study by design. However, the generalizability of the findings remains to be seen in general or clinical population where frequency of cerebrovascular pathology may differ.

In conclusion, this study identified distinct pathways within cerebrovascular pathology whose manifestation is heterogeneous. Arteriolosclerosis and cerebral amyloid angiopathy emerged as dominant markers of this pathology. Greater white matter lesion burden and hippocampal atrophy account for more severe arteriolosclerosis. Decreased white matter microstructural integrity, AD pathology, and cortical atrophy account for more severe cerebral amyloid angiopathy and poorer cognition. The pathway driven by arteriolosclerosis is rather unspecific to AD, while the one driven by cerebral amyloid angiopathy is AD specific. Thus, depending on the etiology, cerebrovascular pathology may act independently of or in tandem with AD pathology, leading to neurodegeneration.

## Supplementary Information

Below is the link to the electronic supplementary material.

Supplementary file1 (DOCX 448 KB)

## Data Availability

This study used data from the Alzheimer's Disease Neuroimaging Initiative (ADNI). All ADNI data are shared through the LONI Image and Data Archive (IDA), a secure research data repository. Interested scientists may obtain access to ADNI imaging, clinical, genomic, and biomarker data for the purposes of scientific investigation, teaching, or planning clinical research studies. Access is contingent on adherence to the ADNI Data Use Agreement and the publication policies outlined in the documents listed below. The application process includes acceptance of the Data Use Agreement and submission of an online application form. The application must include the investigator’s institutional affiliation and the proposed uses of the ADNI. Link to the online repository: https://adni.loni.usc.edu/data-samples/adni-data/.
