# Supplementary material for: Distinct cerebrovascular pathways underlying Alzheimer’s disease-related neurodegeneration
Source: Acta Neuropathol. 2025 Dec 11;150(1):64. doi: 10.1007/s00401-025-02970-8 (PMC12698743; doi:10.1007/s00401-025-02970-8)
Supplement: Supplementary file 1 — Supplementary file1 (DOCX 448 KB) [file 401_2025_2970_MOESM1_ESM.docx]

**Distinct cerebrovascular pathways underlying Alzheimer’s disease-related neurodegeneration**

Rosaleena Mohanty, PhD^1,2^, Sophia Wheatley, MSc^1^, Konstantinos Chiotis, MD, PhD^1^, Anna Marseglia, PhD^1^, Eric Westman, PhD^1,2^, for the Alzheimer’s Disease Neuroimaging Initiative cohort*

^1^ Division of Clinical Geriatrics, Center for Alzheimer Research, Department of Neurobiology, Care Sciences and Society, Karolinska Institutet, Stockholm, Sweden

^2^ The Ageing Epidemiology Research Unit, School of Public Health, Imperial College London, London, UK

**Supplementary Material**

**Supplementary Table 1.** Comparison of infarcts observed on the in vivo MRI and postmortem neuropathologic assessment

| **Number of infarcts on in vivo MRI** | **Presence of infracts in neuropathologic exam** | | |
| --- | --- | --- | --- |
|  | **Old**  **infarcts** | **Old hemorrhages** | **Old microinfarcts** |
| 1 | 1 | 0 | 1 |
| 1 | 0 | 0 | 0 |
| 1 | 0 | 0 | 0 |
| 1 | 0 | 0 | 0 |
| 1 | 0 | 1 | 0 |
| 1 | 0 | 0 | 0 |
| 1 | 0 | 0 | 1 |
| 1 | 0 | 0 | 0 |
| 3 | 0 | 0 | 0 |
| 1 | 1 | 0 | 1 |
| 1 | 0 | 0 | 1 |
| 3 | 0 | 0 | 0 |
| 1 | 0 | 0 | 0 |
| 2 | 0 | 0 | 1 |
| 1 | 0 | 0 | 1 |

**Supplementary Figure 1.** Scree plot showing eigenvalues of multiple correspondence analysis applied to eight cerebrovascular markers


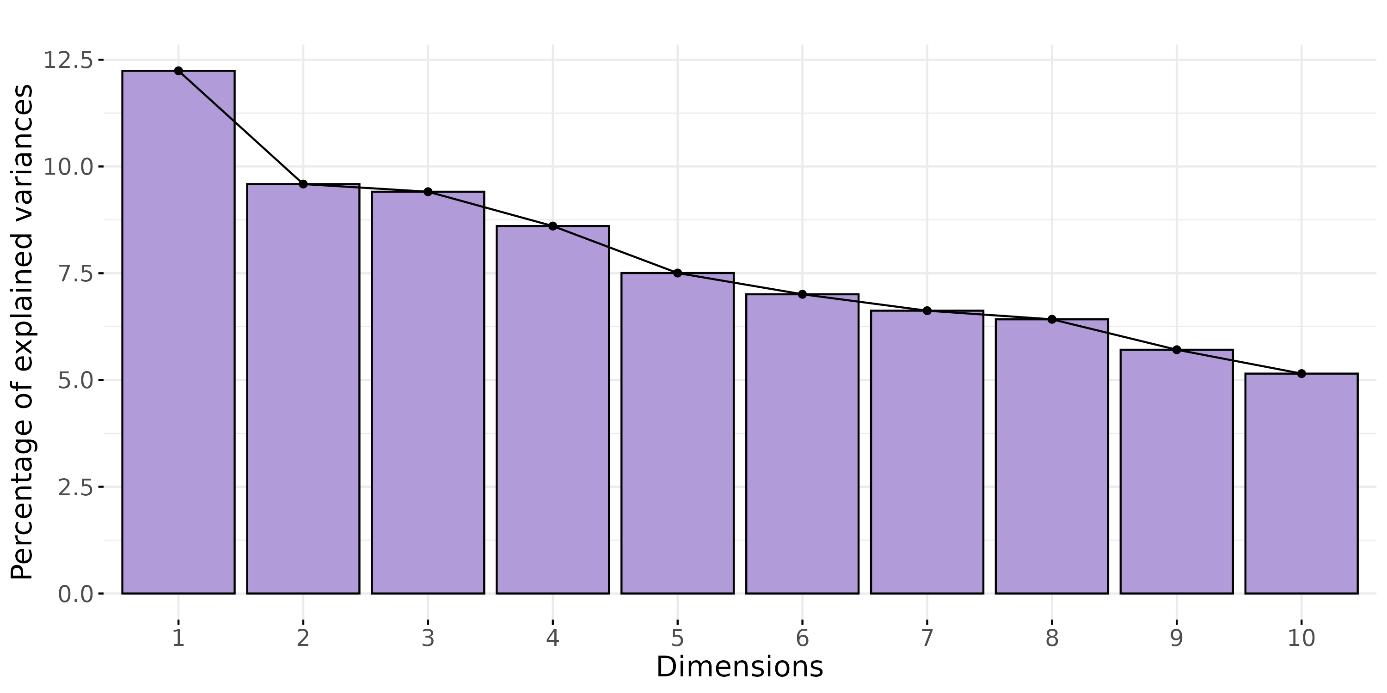


Scree plot shows that the inflection point or “elbow” after component 1 and an incremental gain in going from component 2 to component 3 indicating that the first two dimensions capture the structure in the data while also maintaining the model parsimonious for easier interpretation of the results. Cumulative variance accounted for are 12.2% by dimension 1 and 21.8% by dimensions 1-2.

**Supplementary Table 2.** Ordinal logistic regression showing interaction of white matter hypointensity volume and hippocampal atrophy to explain arteriolosclerosis

| **Outcome: arteriolosclerosis** | ***β*** | **95% confidence interval** | ***p*-value** |
| --- | --- | --- | --- |
| White matter hypointensity ×  hippocampal atrophy | **143.2** | **[63.9, 230.1]** | **0.0003** |
| History of hypertension | -1.2 | [-2.6, 0.05] | 0.06 |
| Age difference (MRI to postmortem) | 0.1 | [-0.006, 0.26] | 0.06 |
| Akaike information criterion | 161.3 | | |

**Supplementary Table 3.** Ordinal logistic regression showing fractional anisotropy and AD pathology to explain cerebral amyloid angiopathy

| **Outcome: cerebral amyloid angiopathy** | ***β*** | **95% confidence interval** | ***p*-value** |
| --- | --- | --- | --- |
| Mean fractional anisotropy | **-20** | **[-41.5, -3.1]** | **0.02** |
| AD neuropathologic change | -0.2 | [-3.0, 2.5] | 0.85 |
| *APOE* ɛ4 carriership | **2.3** | **[0.2, 4.9]** | **0.03** |
| Age difference (DTI to postmortem) | 0.4 | [-0.2, 1.1] | 0.22 |
| Akaike information criterion | 48.5 | | |

**Supplementary Table 4.** Ordinal logistic regression model showing interaction of cortical atrophy and AD pathology to explain cerebral amyloid angiopathy

| **Outcome: cerebral amyloid angiopathy** | ***β*** | **95% confidence interval** | ***p*-value** |
| --- | --- | --- | --- |
| Cortical atrophy ×  AD neuropathologic change | **0.6** | **[0.2, 1.2]** | **0.007** |
| *APOE* ɛ4 carriership | **1.2** | **[0.4, 2.1]** | **0.004** |
| Age at death | -0.002 | [-0.05, 0.05] | 0.93 |
| Akaike information criterion | 227.8 | | |

**Supplementary Table 5.** Multiple linear regression model showing interaction of cerebral amyloid angiopathy and AD neuropathologic change to explain memory scores

| **Outcome: memory composite score** | ***β*** | **95% Confidence interval** | ***p*-value** |
| --- | --- | --- | --- |
| Intercept | -0.7 | [-1.6, 0.2] | 0.12 |
| Cerebral amyloid angiopathy ×  AD neuropathologic change | **-0.2** | **[-0.3, -0.09]** | **0.0009** |
| *APOE* ɛ4 carriership | **-0.3** | **[-0.7, 0.01]** | **0.05** |
| Age difference (cognition to postmortem) | 0.01 | [-0.03, 0.06] | 0.66 |
| Education | 0.02 | [-0.03, 0.07] | 0.4 |
| R^2^ (*p*-value) | **0.23 (<0.0001)** | | |

**Supplementary Figure 2.** In vivo neuroimaging correlates based on MRI, FDG PET and ASL of postmortem arteriolosclerosis and cerebral amyloid angiopathy


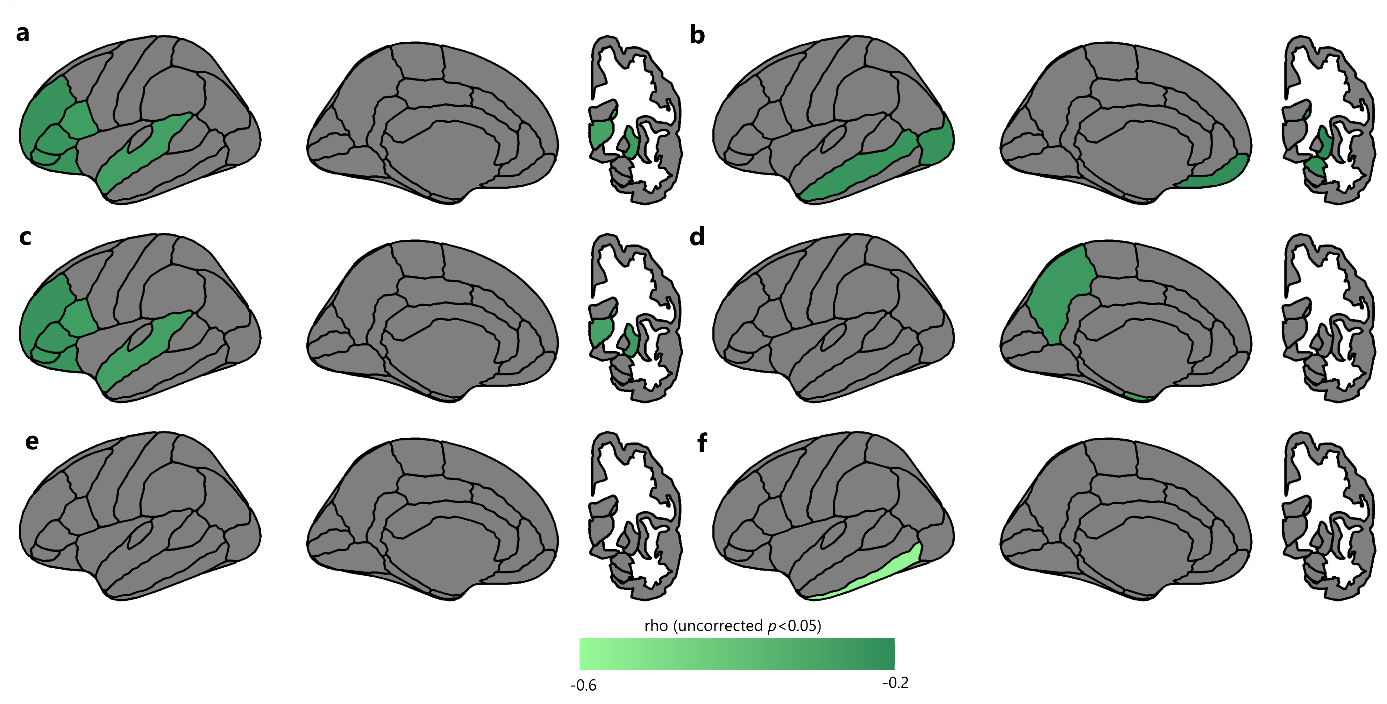


Uncorrected regional associations between postmortem arteriolosclerosis and in vivo **(a)** MRI-based cortical thickness and subcortical volume, **(c)** FDG PET-based standardized uptake value ratio and **(e)** ASL-based cerebral blood flow. Regional association between postmortem cerebral amyloid angiopathy and in vivo **(b)** MRI-based cortical thickness and subcortical volume, **(d)** FDG PET-based standardized uptake value ratio and **(f)** ASL-based cerebral blood flow. Regional values were averaged across left and right hemispheres. Partial correlations were adjusted for neuroimaging-to-postmortem interval and represented by rho for uncorrected *p* < 0.05.
